# CD8^+^ Regulatory T Cell – A Mystery to Be Revealed

**DOI:** 10.3389/fimmu.2021.708874

**Published:** 2021-08-18

**Authors:** Shruti Mishra, Saranya Srinivasan, Chaoyu Ma, Nu Zhang

**Affiliations:** Department of Microbiology, Immunology and Molecular Genetics, The Joe R. and Teresa Lozano Long School of Medicine, University of Texas Health Science Center at San Antonio, San Antonio, TX, United States

**Keywords:** CD8^+^ Treg, transforming growth factor-b, eomes, germinal center, virtual memory T cells, senescence

## Abstract

Regulatory T cells (Treg) are essential to maintain immune homeostasis and prevent autoimmune disorders. While the function and molecular regulation of Foxp3^+^CD4^+^ Tregs are well established, much of CD8^+^ Treg biology remains to be revealed. Here, we will review the heterogenous subsets of CD8^+^ T cells have been named “CD8^+^ Treg” and mainly focus on CD122^hi^Ly49^+^CD8^+^ Tregs present in naïve mice. CD122^hi^Ly49^+^CD8^+^ Tregs, which depends on transcription factor Helios and homeostatic cytokine IL-15, have been established as a non-redundant regulator of germinal center (GC) reaction. Recently, we have demonstrated that TGF-β (Transforming growth factor-β) and transcription factor Eomes (Eomesodermin) are essential for the function and homeostasis of CD8^+^ Tregs. In addition, we will discuss several open questions regarding the differentiation, function and true identity of CD8^+^ Tregs as well as a brief comparison between two regulatory T cell subsets critical to control GC reaction, namely CD4^+^ T_FR_ (follicular regulatory T cells) and CD8^+^ Tregs.

## Introduction

Apart from eliminating specific pathogens and the development of memory B and T cells, another remarkable function of acquired immune response is to differentiate between self and non-self-antigens. This is achieved by educating lymphocytes not to respond to self-antigens and removal of autoreactive cells. This process of unresponsiveness is referred to as self-immune tolerance. Immune tolerance mechanisms ensure that lymphocytes that can mediate a response to the host’s own body are eliminated without impacting the immunogenicity of cells to infection and other foreign antigens. However, despite strict vigilance, checkpoints, and selection process some autoreactive lymphocytes manage to escape and enter the periphery. Regulatory T cells (Treg) are essential regulator of immune tolerance and prevent autoimmunity. In contrast to well-established CD4^+^Foxp3^+^ Tregs (1), recent advances have started to reveal the biology of CD8^+^ Tregs.

## CD8^+^ Treg – A Broad Term Covering Various CD8^+^ Subsets

Various CD8^+^ T cell subsets carrying certain regulatory properties have been discovered in different experimental systems and have been named “CD8^+^ Tregs” (**Table 1**). For example, CD8^+^Foxp3^+^ Tregs have been identified in tissue transplantation and alloantigen-induced immune responses (2–7). CD8^+^CD103^+^ cells are induced from naïve CD8^+^ T cells by short-term *in vitro* stimulation in the presence of TGF-β and exhibit suppressive functions when transferred *in vivo* (8, 9). Further, CD8^+^CD103^+^ cells have been reported in an aggressive tumor model and express Foxp3 (10). However, these cells are often undetectable or extremely rare in unmanipulated mice under steady states. CD8^+^CD28^-^ T cells, carrying suppressive activities exhibit age-dependent accumulation in human (11–14). Similar to CD8^+^Foxp3^+^ cells, CD8^+^CD28^-^ subset is often associated with chronic antigen exposure and does not represent a major population in naïve young adults. Moreover, a sub-population of CD8^+^CD122^+^CD49d^+^ cells express both PD-1 and IL-10, and suppress alloantigen-induced transplant rejection (15–18). To be noted, CD49d expression has been used to identify T cells with prior TCR stimulation (21). Thus, although all these CD8^+^ regulatory T cell populations bear great therapeutic potential, they do not represent a major Treg subset in the absence of overt immune challenge. Importantly, even though CD8^+^CD122^+^CD49d^+^ cells share CD122 expression with CD8^+^CD122^hi^Ly49^+^ CD8 Treg (see discussion below), there is no evidence to support that CD8^+^ T cells can differentiate from one subset to the other. In the following sections, we will mainly focus on CD8^+^CD122^hi^Ly49^+^ CD8 Treg, present in young naïve mice under homeostatic settings.

**Table 1:** Major “CD8^+^ Treg” subsets.

Subset	Precursor	Differentiation condition	Present in young naïve mice	Function	Ref
CD8^+^Foxp3^+^	Mature naïve CD8^+^ T cells (?)	Alloantigen transplantation	None to low	Suppress effector T cell responses and immune rejection	(2–7)
CD8^+^CD103^+^ (vitro)	Mature naïve CD8^+^ T cells	*In vitro* TCR stimulation with TGF-β	None	Suppress immune rejection	(8, 9)
CD8^+^CD103^+^ (tumor)	Mature naïve CD8^+^ T cells	*In vivo* tumor growth	None	Negatively correlated with tumor control	(10)
CD8^+^CD28^-^	Mature naïve CD8^+^ T cells (?)	Age-dependent accumulation	None	Suppress effector T cell responses	(11–14)
CD8^+^CD122^+^ CD49d^+^	Mature naïve CD8^+^ T cells (?)	*In vivo* TCR stimulation	Low	Suppress immune rejection	(15–18)
CD8^+^CD122^+^ Ly49^+^	unknown	Unknown *in vivo* differentiation process	Present	Suppress GC reaction	(19, 20)

## CD122^hi^Ly49^+^CD8^+^ Treg – Discovery and Function

The discovery of these CD8^+^ Tregs is intertwined with the history of suppressor T cells (22). Initial work by Gershon and Cantor demonstrated Qa-1 dependent CD4 suppression by CD8 cells *in vitro* (23–25). This was further confirmed by others studying CD8^+^ T cells in experimental autoimmune encephalomyelitis (EAE) model (26, 27). Later, a series of investigations of non-classical MHC-I molecule Qa-1-restricted T cells has led to the discovery that the regulatory activity of CD8^+^ T cells is largely limited to a subset of memory-like CD8^+^ T cells co-expressing CD122^hi^ and NK marker Ly49 in mice (19, 28–30). Importantly, these CD8^+^ Tregs do not express Foxp3, but instead depend on transcription factor Helios (encoded by *Ikzf2*) (31). Ly49 represents a collection of NK surface receptors (including inhibitory receptors Ly49A, Ly49C/I, Ly49F and Ly49G as well as activating receptor Ly49D and Ly49H). At functional level, but not at sequence level, mouse Ly49 gene cluster is homologous to human KIR (killer-cell immunoglobulin-like receptor) genes (32). On CD8^+^ Tregs, only inhibitory receptors Ly49F, Ly49A, Ly49G and Ly49C/I are detectable (29). All these discoveries together demonstrate that a Qa-1-restricted subset of memory-like CD8^+^ T cells exhibits potent regulatory activity, most likely *via* suppressing or eliminating activated CD4^+^ T cells (33).

Recently, it has been shown that under autoimmunity EAE setting, CD8^+^CD122^hi^Ly49^+^ Tregs are not Qa-1-restricted and can be derived from classical MHC-I-restricted CD8^+^ T cells (34). This work also demonstrates that immune activation induced CD8^+^ Tregs may be a general player required for immune homeostasis. Importantly, these immune activation-induced CD8^+^ Tregs bear gene expression signature similar to Qa-1 restricted CD8^+^ Tregs (34).

In a separated line of research, it has been discovered that CD122^hi^CD8^+^ T cells suppress lymphopenia-induced T cell proliferation and autoimmunity in CD122 deficient mice (35). Putting all these elegant works together, it becomes clear that in naïve mice, a subset of both non-classical MHC-I molecule Qa-1-dependent and classical MHC-I-dependent CD8^+^CD122^hi^Ly49^+^ T cells express Helios and suppress autoimmunity. Upon immune activation, these CD8^+^ Tregs further respond to activation-associated epitopes on CD4^+^ T cells to fine tune ongoing immune responses.

In addition to abovementioned EAE model (19, 36, 37), CD8^+^CD122^hi^Ly49^+^ Tregs have documented functions in various settings, including colitis (38, 39), hepatitis (40), arthritis (41), diabetes (42, 43), viral infection (44), tumor immunity (45), atherogenesis (46) and organ transplantation (47). The best demonstrated role of CD8^+^ Tregs is to suppress GC reaction. Multiple different animal models with defective CD8^+^ Tregs exhibit enhanced GC reaction and autoantibody production (20, 28, 31, 48), suggesting a non-redundant role for CD8^+^ Tregs to mitigate GC response.

## CD122^hi^Ly49^+^CD8^+^ Treg – Effector Molecules

IL-10 was dispensable for CD8^+^ Treg regulatory action as anti-IL10 antibody treatment did not alter CD8^+^ Treg function (28). CD8^+^ T cells are considered cytotoxic. CD8^+^ Tregs perform their regulatory function by killing antigen activated CD4^+^ T cells and T_FH_ cells. The main cytotoxic molecule shown to be important for CD8^+^ Treg function is perforin. CD8^+^ Treg from *Prf1^-/-^* mice were not able to suppress antigen-activated T_FH_ cells in *Rag2^-/-^* mice (28). In another study done in EAE mice model, CD8^+^ Tregs from *Prf1^-/-^* mice were unable to suppress the proliferation of antigen-activated CD4^+^ T cells further confirming the importance of perforin in CD8^+^ Treg mediated suppression (34). However, more studies need to be performed to better understand the mechanism of perforin action. For example, our recent findings have demonstrated that WT CD8^+^ Tregs do not express granzyme A and granzyme B under steady states (20). It will be interesting to examine whether the expression of granzyme A and granzyme B is only induced upon detection of auto-reactive targeting cells or other granzyme(s) are involved in the function of CD8^+^ Tregs.

In addition, FasL has been demonstrated to be required for CD8^+^ Tregs to suppress activation and proliferation of both CD4^+^ and CD8^+^ T cells (49). Importantly, Fas and FasL signals are essential controllers of CD8^+^ Treg homeostasis as both *lpr* and *gld* mice carry an increased number of CD8^+^ Tregs. However, whether Fas/FasL directly affects CD8^+^ Tregs or indirectly impacts CD8^+^ Tregs *via* other immune cells remain uninvestigated. To be noted, similar to other effector T cells, TCR engagement is essential for CD8^+^ Tregs (34, 49). Together, accumulating evidence has demonstrated that upon cognate antigen encounter, CD8^+^CD122^hi^Ly49^+^ Tregs target activated CD4^+^ T cells *via* a perforin and/or Fas/FasL dependent mechanism to suppress ongoing GC reaction.

## CD122^hi^Ly49^+^CD8^+^ Treg – Molecular Regulators

Not surprisingly, IL-15 signal is essential for CD8^+^ Treg homeostasis as they carry high levels of IL-15 receptor CD122 (37). Regulatory T cell associated transcription factor Helios is essential for the function of CD8^+^ Treg, partially *via* controlling IL-15/STAT5 pathway (31). Cytoskeleton regulator moesin controls CD8^+^ Tregs *via* regulating IL-15 receptor internalization (48). Further, boosting IL-15 signal is sufficient to expand CD8^+^ Tregs and suppress autoimmune diabetes (42).

We recently discovered a lethal autoimmune phenotype in a T cell-specific conditional knockout mice line lacking both TGF-β receptor and transcription factor Eomes (20). In these double knockout (DKO) mice, greatly enhanced spontaneous GC reaction and autoantibody production are tightly associated with gradual decrease and eventually disappearance of CD8^+^ Treg population. Importantly, CD4^+^ Treg compartment is largely intact in DKO mice. In addition, we discover that TGF-β signal and Eomes play critical roles in three seemingly different aspects of CD8^+^ Treg biology. First, TGF-β signal is essential for the induction of Helios and suppression of effector molecules (e.g., granzyme A, granzyme B and KLRG-1). Second, Eomes controls a molecular program critical for the follicular location and migration of CD8^+^ Tregs, which includes the induction of CXCR5 and inhibition of integrins α1 and α4. Importantly, these functions of Eomes are not shared with the closely related transcription factor T-bet. Finally, TGF-β and Eomes coordinate to promote the expression of IL-15 receptor and pro-survival molecule Bcl-2. Further, the abovementioned functions of TGF-β and Eomes are CD8^+^ T cell-specific without impacting CD4^+^ T cells including CD4^+^ Tregs. Thus, our findings may establish DKO mice as a valuable CD8^+^ Treg deficient model with an intact CD4^+^ Treg compartment (50).

Strikingly, our results also reveal that CD8^+^ Tregs are extremely potent suppressors of autoimmunity (20). Adoptive transfer of a small number of WT CD8^+^ Treg into DKO mice is sufficient to fully suppress the spontaneous GC reaction without fully reconstituting the CD8^+^ Treg compartment. In both TGF-βR single knockout and Eomes single knockout mice, even though CD8^+^ Treg population is significantly reduced, no overt autoimmunity is detected. Thus, even a small portion of CD8^+^ Tregs is sufficient to mitigate autoimmunity at steady states, which imply significant translational potential for treating human autoimmune disorders.

## Open Questions

### Why Does GC Need Two Distinct Regulatory T Cell Subsets?

As we have discussed that the best-established function of CD8^+^CD122^hi^Ly49^+^ Tregs is to suppress GC reaction. In addition to these CD8^+^ Tregs, a long line of literature has suggested the suppressive function of CD4^+^Foxp3^+^T_FR_ (Foxp3^+^ follicular regulatory T cell) in dampening GC reactions. An obvious question appears–why does GC need two distinct regulatory T cell subsets? In this section, we would like to briefly summarize the recent findings in T_FR_ research and present a side-by-side comparison of CD8^+^CD122^hi^Ly49^+^ Tregs with CD4^+^ T_FR_s (**Table 2**).

**Table 2:** T_FR_ and CD8^+^CD122^+^Ly49^+^ comparison.

	T_FR_	CD8^+^CD122^+^Ly49^+^
T cell subset	CD4^+^	CD8^+^
Regulatory identity-TF	Foxp3^+^ (Helios^+/-^?)	Foxp3^-^Helios^+^
Other TF	Bcl6^+^	Eomes^+^
Common surface marker	CXCR5^+^	CXCR5^+^
Mitigate spontaneous GC/autoimmunity	Mostly no	Yes
Effector molecules	IL-10, neuritin	Perforin, FasL
Function in GC	Promote GC during viral infection and peanut allergic responses; Suppress early GC following protein immunization and HDM allergic responses.	Suppress GC
Targeting cells	CD4^+^ and B	CD4^+^

To specifically targeting CD4^+^ T_FR_ without affecting other CD4^+^Foxp3^+^ Tregs, several elegant genetic models have been developed. First, a *Bcl6* ^f/f^Foxp3-Cre mouse line has been demonstrated to lack T_FR_ cells. Only subtle and late onset autoimmunity has been reported for this T_FR_-deficient mouse line (51). However, upon immunization there is decreased antigen-specific IgG and increased IgA response along with decreased antigen-specific antibody avidity (52). During influenza viral infection, T_FR_ either has no impacts (51, 53) or promotes antigen-specific B cell responses (54) *via* an IL-10-dependent mechanism (55). Similarly, in a peanut allergy model, T_FR_ cells help maintain GC and antigen-specific IgE response in an IL-10 dependent manner (56).

In a different model, where the expression of CXCR5 is deleted on Foxp3^+^ cells in an inducible manner, the reduction of T_FR_ leads to no alteration in GC reaction or antibody production (57). All these data together suggest that T_FR_ cells either have very subtle impacts or more of a helper function to promote antigen-specific GC response.

In an inducible T_FR_-deleter mouse line, it is shown that T_FR_ selectively suppresses early GC response and spares later GC response after protein immunization (58). House dust mite (HDM) induced Th2 response is greatly enhanced in this T_FR_-deleter mice (58), which is consistent with another report using *Bcl6* ^f/f^Fxop3-cre mice and HDM challenge model (59). A recent publication using *Bcl6* ^f/f^Fxop3-cre mice identifies a key effector molecule neuritin used by T_FR_ to directly inhibit GC B cells, especially under IgE skewing Th2 condition (60). Taken together, considerable discrepancy exists regarding the *in vivo* function of T_FR_. In addition to the intrinsic difference between various genetic models (e.g., *Bcl6* ^f/f^Foxp3-Cre may have defects in other Foxp3^+^ cells and CXCR5 deletion may not eliminate all T_FR_-associated activity), the difference between immune challenge models may be critical. For example, while T_FR_ only target early GC reaction (day 5-9) following protein immunization (58), the number of T_FR_ significantly increases only late (>day 30) after influenza viral infection (53, 54). Th2 allergic response provides a distinct immune environment from viral infection. Food allergy is different from HDM allergy (e.g., targeting tissues). Different immune environment may create different niche for T_FR_, which may correlate with their various demonstrated functions.

In contrast to CD4^+^T_FR_, CD8^+^ Treg is generally considered as immune suppressor. To answer the question why GCs need two subsets of regulatory T cells, both population kinetics of CD4^+^ T_FR_ *vs* CD8^+^ Tregs and various immune challenging conditions need to be taken into consideration. A significant number of CD8^+^ Tregs (around 0.3x10^6^ cells/spleen) is present before immune challenge in secondary lymphoid organs. We have recently found that CD8^+^ Treg population is significantly reduced in response to an autoimmune stimulus (20). In contrast, T_FR_-deficient mice do not exhibit severe autoimmunity in the absence of challenge. Thus, CD8^+^ Treg may be the critical gate keeper of spontaneous GC reaction at steady states. Depending on the nature of immune stimuli, either CD8^+^ Treg or CD4^+^ Treg or both will be required to keep the fine balance between an effective immune response and excess tissue damage/autoimmunity.

### What Is the True Identity of CD8^+^ Treg?

In spite of commonly expressed Treg associated transcription factor Helios, unbiased transcriptional analysis suggests that CD8^+^ Tregs are closely resemble antigen-experienced CD8^+^ T cells, but distinct from CD4^+^ Tregs (20). While investigating the lineage relationship between CD122^hi^Ly49^+^CD8^+^ Tregs and other CD8^+^ T cells, two prominent features of CD8^+^ Tregs are hard to ignore.

First, CD8^+^ Tregs bear similar markers of senescent T cells. Studies in human have identified age-dependent accumulation of CD28^-^CD8^+^ T cells carrying certain regulatory activities (11–14). Even though the relationship between CD28^-^CD8^+^ and CD122^hi^Ly49^+^ CD8^+^ Tregs is not clear at present, it has been shown that CD27^-^CD28^-^CD8^+^ senescent T cells carries increased levels of NK-associated markers, including Ly49 in mice (61). Further, CD8^+^ Treg gene signature has been established as a reference of dysfunctional CD8^+^ T cells during chronic antigen exposure (62). A few more pieces of evidence include age-dependent accumulation of Ly49^+^CD8^+^ T cells in mice (61, 63, 64) and age-dependent reduction of regulatory activity of CD122^hi^CD8^+^ T cells (35). Together, all the evidence points to an interesting relationship between CD8^+^ Tregs and age-dependent accumulation of senescent T cells.

Secondly, virtual memory T cells (T_VM_) represent a subset of memory-like CD8^+^ T cells differentiated *via* homeostatic proliferation without previous cognate antigen encounter (65–67). T_VM_ cells bear similar markers as CD8^+^ Tregs (e.g., CD122^hi^ and CD49d^lo^). At molecular levels, IL-15 is essential for T_VM_ homeostasis (68). TGF-β signal controls T cell homeostatic proliferation (69, 70), therefore most likely impacts T_VM_ differentiation. Eomes is required for T_VM_ differentiation (71). All of these molecular regulators control both T_VM_ and CD8^+^ Treg. The only known surface marker to distinguish T_VM_ *vs* CD8^+^ Treg is Ly49. Recently, using an Eomes-reporter mouse line, a thymic precursor for T_VM_ has been identified (72). Whether a subset of these Eomes^+^ precursors will differentiate into CD8^+^ Tregs remains an open question. It remains to be established what are the differential signals control the differentiation of T_VM_ *vs* CD8^+^ Treg.

## Conclusion

Significant progress has been made in CD8^+^ Treg field in recent decades. Both Qa-1-restricted and classical MHC-I-restricted CD8^+^ Tregs bearing surface CD122 and Ly49 are present in young naïve mice. These CD8^+^ Tregs play a non-redundant function to mitigate spontaneous GC reaction and various immune responses *via* suppressing activated CD4^+^ T cells. Transcription factor Helios is required for the regulatory function of CD8^+^ Tregs while Eomes is essential for their follicular location. Cytokine IL-15 controls their homeostasis and TGF-β is required to maintain their regulatory identity. In spite of these advances, open questions regarding their true identity and differentiation/development path remain to be addressed in the future. Further, even though both our group and others have provided evidence that a similar human CD8^+^ Treg subset does exist (20, 43), due to the facts that Ly49 is a mouse-specific gene cluster without human homologs at sequence level, human CD8^+^ Treg remains to be firmly established. Future investigation into CD8^+^ Treg will not only provide an opportunity to design future therapeutic interventions for autoimmune disorders, but will also elucidate a mysterious path of CD8^+^ T cell differentiation.

## Author Contributions

SM and NZ researched and wrote the manuscript. SS and CM edited the manuscript. All authors contributed to the article and approved the submitted version.

## Funding

This work is supported by NIH grant AI125701, American Cancer Society grant RGS-18-222-01-LIB, a W.M. Keck Foundation grant and by funds from the UT Health San Antonio to NZ.

## Conflict of Interest

The authors declare that the research was conducted in the absence of any commercial or financial relationships that could be construed as a potential conflict of interest.

## Publisher’s Note

All claims expressed in this article are solely those of the authors and do not necessarily represent those of their affiliated organizations, or those of the publisher, the editors and the reviewers. Any product that may be evaluated in this article, or claim that may be made by its manufacturer, is not guaranteed or endorsed by the publisher.
